# MR Imaging of Peripheral Nerves Using Targeted Application of Contrast Agents: An Experimental Proof-of-Concept Study

**DOI:** 10.3389/fmed.2020.613138

**Published:** 2020-12-11

**Authors:** Vlad Tereshenko, Irena Pashkunova-Martic, Krisztina Manzano-Szalai, Joachim Friske, Konstantin D. Bergmeister, Christopher Festin, Martin Aman, Laura A. Hruby, Johanna Klepetko, Sarah Theiner, Matthias H. M. Klose, Bernhard Keppler, Thomas H. Helbich, Oskar C. Aszmann

**Affiliations:** ^1^Clinical Laboratory for Bionic Extremity Reconstruction, Division of Plastic and Reconstructive Surgery, Department of Surgery, Medical University of Vienna, Vienna, Austria; ^2^Center for Biomedical Research, Medical University of Vienna, Vienna, Austria; ^3^Department of Biomedical Imaging and Image-guided Therapy, Division of Molecular and Structural Preclinical Imaging, Medical University of Vienna & General Hospital, Vienna, Austria; ^4^Institute of Inorganic Chemistry, University of Vienna, Vienna, Austria; ^5^Centre for Microbiology and Environmental Systems Science, University of Vienna, Vienna, Austria; ^6^Department of Plastic, Aesthetic and Reconstructive Surgery, University Hospital St. Poelten, Karl Landsteiner University of Health Sciences, Krems, Austria; ^7^Department of Hand, Plastic and Reconstructive Surgery, Burn Center, BG Trauma Hospital Ludwigshafen, University of Heidelberg, Heidelberg, Germany; ^8^Department of Orthopedics and Trauma Surgery, Medical University of Vienna, Vienna, Austria; ^9^Division of Plastic and Reconstructive Surgery, Medical University of Vienna, Vienna, Austria

**Keywords:** peripheral nerve, spinal cord, axonal transport, contrast agents, MRI, nerve injury, nerve repair

## Abstract

**Introduction:** Current imaging modalities for peripheral nerves display the nerve's structure but not its function. Based on a nerve's capacity for axonal transport, it may be visualized by targeted application of a contrast agent and assessing the distribution through radiological imaging, thus revealing a nerve's continuity. This concept has not been explored, however, may potentially guide the treatment of peripheral nerve injuries. In this experimental proof-of-concept study, we tested imaging through MRI after administering gadolinium-based contrast agents which were then retrogradely transported.

**Methods:** We synthesized MRI contrast agents consisting of paramagnetic agents and various axonal transport facilitators (HSA-DTPA-Gd, chitosan-DTPA-Gd or PLA/HSA-DTPA-Gd). First, we measured their relaxivity values *in vitro* to assess their radiological suitability. Subsequently, the sciatic nerve of 24 rats was cut and labeled with one of the contrast agents to achieve retrograde distribution along the nerve. One week after surgery, the spinal cords and sciatic nerves were harvested to visualize the distribution of the respective contrast agent using 7T MRI. *In vivo* MRI measurements were performed using 9.4 T MRI on the 1st, 3rd, and the 7th day after surgery. Following radiological imaging, the concentration of gadolinium in the harvested samples was analyzed using inductively coupled mass spectrometry (ICP-MS).

**Results:** All contrast agents demonstrated high relaxivity values, varying between 12.1 and 116.0 mM^−1^s^−1^. HSA-DTPA-Gd and PLA/HSA-DTPA-Gd application resulted in signal enhancement in the vertebral canal and in the sciatic nerve in e*x vivo* MRI. *In vivo* measurements revealed significant signal enhancement in the sciatic nerve on the 3rd and 7th day after HSA-DTPA-Gd and chitosan-DTPA-Gd *(p* < 0.05) application. Chemical evaluation showed high gadolinium concentration in the sciatic nerve for HSA-DTPA-Gd (5.218 ± 0.860 ng/mg) and chitosan-DTPA-Gd (4.291 ± 1.290 ng/mg).

**Discussion:** In this study a novel imaging approach for the evaluation of a peripheral nerve's integrity was implemented. The findings provide radiological and chemical evidence of successful contrast agent uptake along the sciatic nerve and its distribution within the spinal canal in rats. This novel concept may assist in the diagnostic process of peripheral nerve injuries in the future.

## Introduction

Peripheral nerve injuries may result from traumatic events, compressional or inflammatory pathologies and iatrogenic injuries (1, 2). If left untreated, these lesions may manifest in severe motor and/or sensory deficits, which cause significant health care costs and can severely reduce a patient's quality of life (3–5). The therapeutic management of peripheral nerve lesions requires detailed information about the exact localization and extent of an injury, especially in closed traumata (6).

Generally, the diagnosis of peripheral nerve injuries or peripheral neuropathies is based on medical history, comprehensive clinical examination and electrophysiological tests combined with specific imaging techniques (7). The gold-standard for the functional analysis of nerve injuries is a detailed clinical examination together with various neurophysiologic tests, which, however, can have limited diagnostic value in terms of grading the injury, especially within the first few weeks after injury (7, 8). Imaging of nerve injuries is done through sonography; however, this modality is limited by the accessibility of deep nerves and potential swelling and early hematoma. Alternatively, magnetic resonance neurography (MNR) allows visualization of nerve tissue alterations and anatomical disruption of peripheral nerves (9–11). However, it is scarcely used due to limited availability of high-resolution magnetic resonance imaging (MRI) scanners and subsequent centers of expertise (12).

Recently, numerous experimental MRI trials in rat models were able to demonstrate contrast enhancement in focal nerve lesions using systemic application of different contrast agents (13–21). These studies focused on dynamic visualization of ongoing Wallerian degeneration or demyelination processes, targeting macrophage transmigration based on blood-nerve barrier leakage (14, 16, 17). This spatiotemporal contrast enhancement does currently not provide reliable information on nerve regeneration and axonal continuity within an injured nerve (22).

In peripheral nerves, molecules and organelles are physiologically carried along an axon's entire length via axonal transport (23, 24). This process can be studied in an experimental setting through retrograde labeling, which is based on the transport of fluorescent dyes from the point of application to the nerve's perikaryon (25, 26). Hence, successful retrograde delivery of substances to neurons in the spinal cord or brainstem indicate functional continuity. Today, there are no diagnostic approaches utilizing axonal transport to assess the functional capacity of peripheral nerves (26).

**Figure 1:**
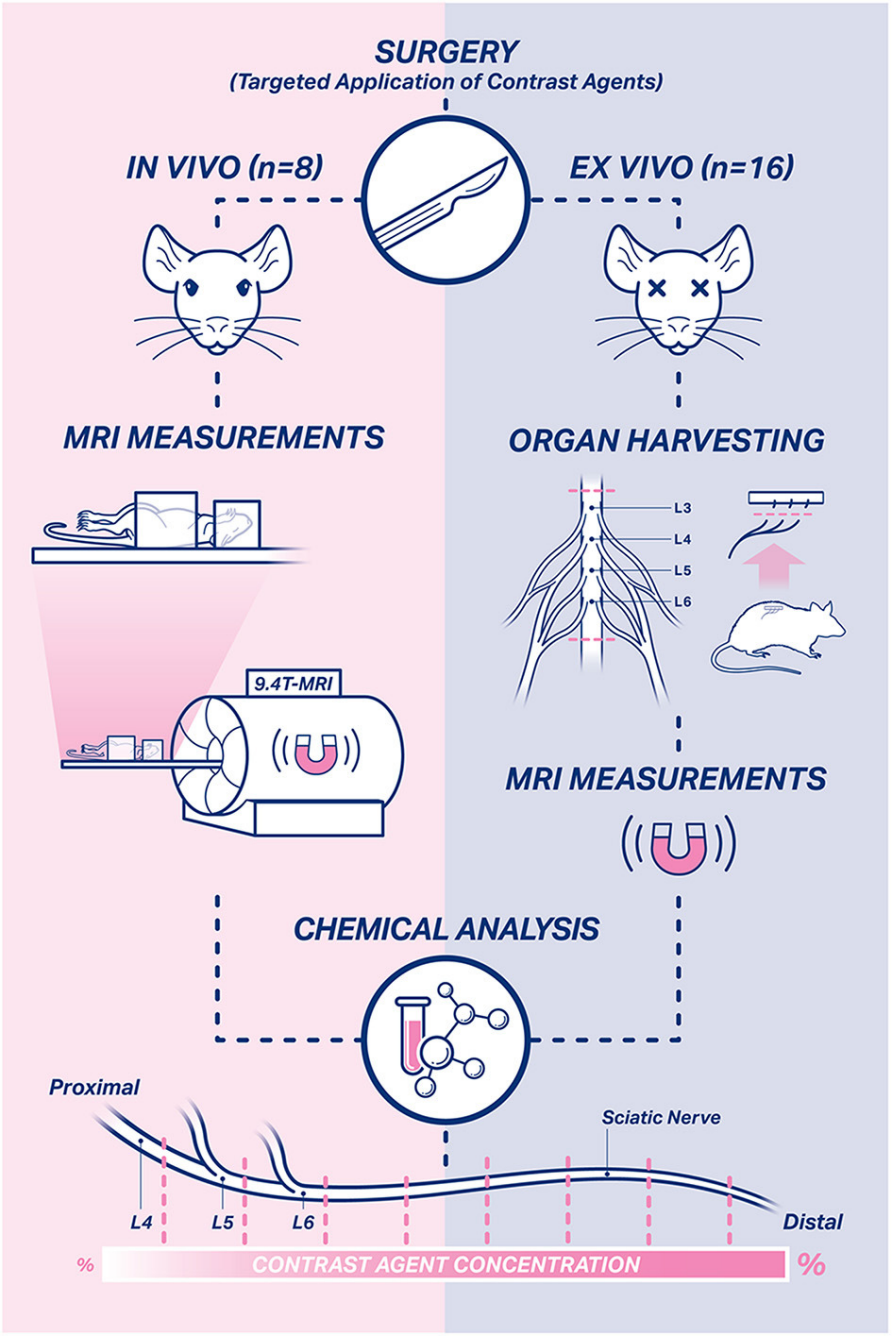
Study design. In total, 24 rats were used in this study. *In vivo* measurements were performed in eight rats on the 1st, 3rd, and 7th day after surgery. *Ex vivo* MRI measurements were performed in harvested sciatic nerve and spinal cord samples of 16 rats on the 7th day after surgery. Afterwards, the harvested nerve and spinal cord samples of all rats were subjected to chemical evaluation to assess the temporospatial propagation of the contrast agents.

In this *proof-of-concept study*, we investigated a novel approach to visualize a peripheral nerve's functional continuity by novel contrast agents for MRI (Figure 1). The contrast agents were produced as macromolecular conjugates comprised of gadolinium (Gd) chelates and specific carriers (human serum albumin, polylactic acid (PLA) and chitosan), which allow retrograde axonal transport (27, 28). First, the contrast agents were tested *in vitro* regarding their suitability for radiological use. This was followed by targeted, local application to the sciatic nerve of rats and subsequent MR imaging. We present radiological and chemical evidence demonstrating successful uptake and retrograde transport of these novel contrast agents along the nerve up to the vertebral cavity.

## Materials and Methods

### Contrast Agents

Different carrier molecules known for their property to be transported by the axonal cytoskeleton were selected for the development of the Gd-based contrast agents (29). Human serum albumin (HSA, fatty acid free, essentially globulin free, lyophilized powder ≥ 99%, molecular weight 66 kDa); polylactic acid (PLA, poly-D,L-lactide, molecular weight 75–120 kDa, a biopolymer) and chitosan oligosaccharides (≥75% deacetylation grade, molecular weight ≤ 5 kDa, Heppe Medical Chitosan GmbH) were used in this study. A detailed synthesis protocol will be published separately.

### *In vitro* Measurements

The three contrast agents were diluted in phosphate-buffered-saline (PBS) to concentrations of 0.1, 0.01, 0.001, 0.001 M. A tube with pure PBS 0.1 M solution was used as a negative control. To evaluate the relaxivity values of the contrast agents, circular tubes (2 ml) were filled with the different dilutions and placed in an Eppendorf storage box. Measurements were performed with a 3 Tesla whole-body MRI scanner (Magnetom PrismaFit 3T, High Field MR Center, Vienna, Austria) using a MRI knee (birdcage type) coil with an inner diameter of 19 mm. The T1 relaxation time constants were determined with inversion recovery sequences (TI) = 15, 30, 60, 100, 200, 300, 400, 500, 700, 800, 900, 1000, 1200, 1300, 1400, 1500, 1600, 1700, 1800, 1900, 2000, 3000, 4000, 5000 ms. The echo time (TE) was 10 ms and the repetition time (TR) was between 12,500 and 15,000 ms. A field of view (FoV) read 480 mm, the FoV phase was 75%, slice thickness was set at 1 mm and the nominal flip angle was 20 degrees. Region of interest (ROI) areas were constructed manually and placed over the cross-sections of the circular tubes filled with the diluted contrast agents. The longitudinal relaxation time was measured within a ROI for each contrast agent at every concentration. The relaxivity values were calculated using the MATLAB and Statistics Toolbox Release 2012b, as described previously (The MathWorks, Inc., Natick, Massachusetts, United States) (30).

### Application of Contrast Agents

All animal experiments were approved by the ethics committee of the Medical University of Vienna and the Austrian Federal Ministry of Science and Research (reference number: BMWF-66.009/0025-WF/V/3b/2017).

In two separate trials, a total of 24 male Sprague-Dawley rats (Department for Laboratory Animal Science and Genetics, Himberg, Austria) aged 8–10 weeks underwent surgical intervention. The animals were housed with a 12-h light-dark cycle, received standard rat chow (Fa Ssniff, Germany) and water ad libitum. All animals received human care in compliance with the principles of laboratory animal care as recommended by FELASA at the Department of Biomedical Science at the Medical University of Vienna.

Preoperative anesthesia and analgesia were performed via intraperitoneal injection of ketamine (100 mg/kg) and xylazine (5 mg/kg). Afterwards, all rats were anesthetized with isoflurane (2%) via a Vaporizer (Draeger Vapor 19.3 Forane®) and then received a subcutaneous injection of piritramide (0.3 mg/kg) for further analgesia.

**Figure 2:**
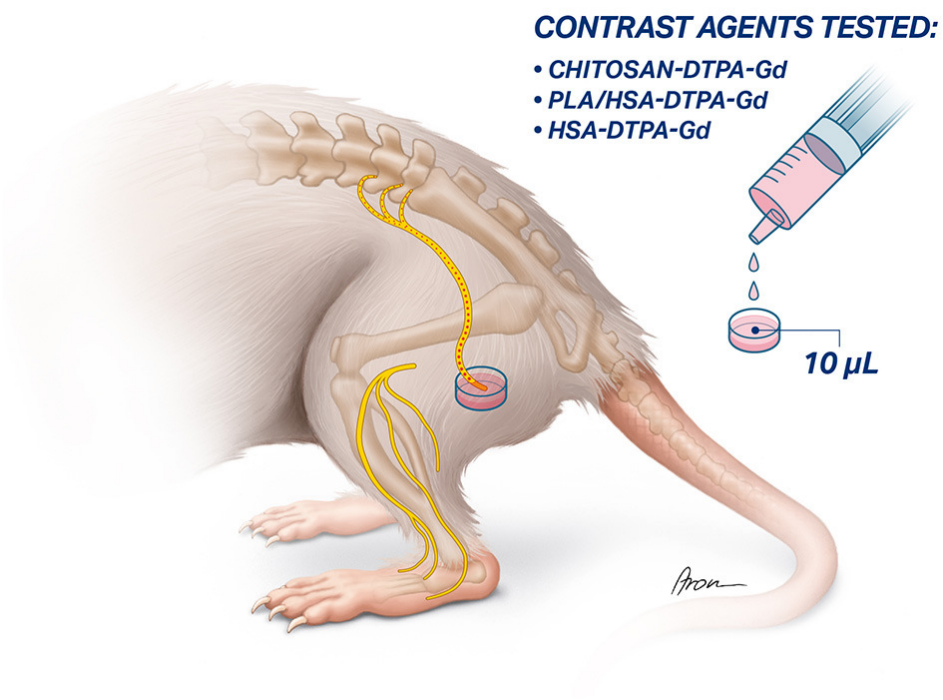
Schematic illustration of targeted application of contrast agents to the sciatic nerve. the sciatic nerve is exposed and transected proximal to its trifurcation. Afterwards, the proximal stump of the sciatic nerve is inserted into a vessel filled up with the 10 μl of the contrast agent and left there for 1 h to allow the absorption and retrograde transport of the contract agent.

The sciatic nerve was exposed between the superficial gluteal and biceps femoris muscle. After identifying the sciatic nerve, the nerve trunk was transected proximal to its trifurcation. The proximal stump was placed into the cap of a PCR tube (0.2 mL, Eppendorf, Switzerland) filled with 10 μl of a contrast agent solution. The proximal nerve stump remained in the contrast agent solution for 1 h to allow sufficient uptake (Figure 2). Afterwards, excess contrast agent was washed out with sterile NaCl (0.9%) solution to prevent undesirable accumulation in the surrounding tissue.

### *Ex vivo* MRI Measurements

The physicochemical behavior of the different contrast agents in nerve tissue was investigated in 16 rats: HSA-DTPA-Gd 0.06 mM (n=4), chitosan-DTPA-Gd 0.076 mM (*n* = 2), PLA/HSA-DTPA-Gd 0.13 mM (*n* = 4), HSA-DTPA-Gd 1.72 mM (*n* = 4) and prohance (*n* = 2) as control.

Seven days after targeted application of the contrast agent, the animals were euthanized and tissue samples harvested. Hereby, the proximal sciatic nerve stump was identified and carefully dissected further proximally to where the four lumbar spinal nerves (L3–L6) unite. Each of them was cut just distal to the intervertebral foramen. The intact contralateral sciatic nerve was harvested and used as control sample. The spinal cord was exposed from segment L1 to L6 via a dorsal approach and harvested as well. The samples were then stored at +4°C for 24 h. Four thawed sciatic nerves were then placed each into one of the four chambers created by a 10 ml concentric syringe's barrel and its cross-shaped plunger filled with 0.1 M PBS solution. It was then blocked up on both ends to prevent the nerves from drying out. Two nerve samples were also placed in a syringe parallel with tubes filled with prohance 25 mM to compare the signal enhancement. The spinal cords were placed into a 10 ml syringe filled with 0.1 M PBS.

*Ex vivo* measurements were performed using a 7 Tesla MRI scanner (Magneton 7T, High Field MR Center, Vienna, Austria) with a high-resolution MR-microimaging gradient insert (maximal gradient strength 750 mT/m) and a T1 inversion recovery (T1IR) sequence. The imaging protocol for the nerve samples started with localizer scans followed by the inversion recovery T1 weighted sequence (TR = 12,500 ms, TE = 5.8 ms, TI = 1,500–13,000 ms, 78 μm in plain resolution) in the transverse plane with a slice thickness of 1 mm. The imaging protocol for the spinal cord consisted of inversion recovery T1 weighted sequences (TR = 16,000 ms, TE = 5.8 ms, TI = 100–3,500 ms, 78 μm in plain resolution) in the transverse and sagittal plane with a slice thickness of 1 mm. The acquisition time was approximately 5 min per inversion recovery sequence and 1 h in total per sample. MATLAB (The MathWorks, Inc., Natick, Massachusetts, United States) was used to calculate T1 maps via a least squares method.

### *In vivo* MRI Measurements

*In vivo* MRI measurement were performed in 8 rats. Two were operated without using a contrast agent, as described above. The first measurement was carried out 1 day before surgery. Following intraoperative labeling with HSA-DTPA-Gd 0.06 mM (*n* = 2), PLA/HSA-DTPA-Gd 0.13 mM (*n* = 2) and chitosan-DTPA-Gd 0.076 mM (*n* = 2), measurements were performed on the 1st, 3rd, and 7th day after surgery to identify propagation of the respective contrast agent along the nerve.

All *in vivo* MR scans were obtained with a 9.4 Tesla MRI scanner (Biospec 94/30, Bruker, Ettlingen, Germany) with a 30 cm bore and a BGA12S gradient system (maximal gradient strength 667 mT/m, 12 cm bore). All animals were anesthetized with a gas mixture (30% oxygen and 1.5–2% isoflurane) using a rat face mask and an isoflurane vaporizer (Vapor 19.3 Forane®, Draeger, Luebeck, Germany) during all measurements. No muscle relaxant was administered. The rats were placed in a supine position on an integrated multimodal animal bed. Furthermore, heart rate, respiratory rate and oxygen saturation were monitored during the entire scanning procedure.

For the *in vivo* experiments, multi-slice T2-weighted images were acquired for anatomical orientation using a “rapid acquisition with relaxation enhancement” (RARE) sequence to minimize the acquisition time. T1-weighted images were obtained to depict the signal enhancement. The imaging protocol was established in advance in independent experiments with unexposed euthanized rats. The following imaging parameters were used for the T1-weighted images: TR = 415 ms, TE = 6.89 ms, acquisition time = 105 s; while these parameters were used for T2-weighted images: TR = 3,763 ms, TE = 23 ms, acquisition time = 112 s. MRI scans were acquired in the sagittal, transverse and coronal plane.

**Figure 3:**
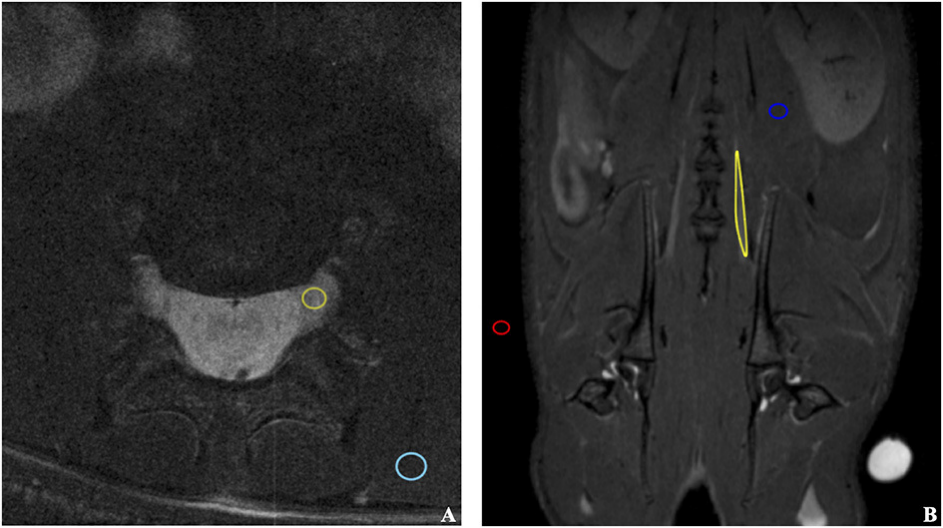
Illustration of ROIs selection on coronal **(A)** and transverse **(B)** MRI scans. The ROIs were selected for the quantitative evaluation of the signal enhancement in the sciatic nerve exposed to the contrast agent and other tissues. The following structures were marked: background air (red), unaffected muscle (blue), sciatic nerve (yellow).

MRI scans were processed using manual ROI selection for the sciatic nerve, spinal nerves (L5/L6), unaffected muscle tissue and background air (Figure 3). ROIs were selected in the T1WI scans based on the aligned T2WI scans. The signal-to-noise ratio (SNR) was calculated in each ROI as a quotient of the mean ROI signal intensity to the standard deviation (SD) of the background noise (31). Signal enhancement increase due to intraoperative contrast agent application was measured as the percentage increase of the SNR in the nerve and muscle tissue. Calculated signal enhancement increase was presented as mean values and standard deviation. In this small sample size we assumed normal distribution of our data. Furthermore, signal enhancement increase in the sciatic nerve after contrast agent application was compared to the native contralateral sciatic nerve, the transected sciatic nerve without any contrast agent application as well as to the unaffected muscle using a 2-tailed, 2-sample Student's *t*-test and the significance level was set at *p* = 0.05.

### Chemical Evaluation

Following the MRI measurements, the nerve and spinal cord samples were subjected to chemical analysis to assess Gd uptake by the sciatic nerve. Excised nerves were cut into 0.5 cm long segments (Figure 1). The Gd concentration of each nerve segment and spinal cord was determined by ICP-MS. A detailed description of this method will be published separately.

## Results

### Efficacy of the Novel Contrast Agents

The newly synthesized contrast agents were first analyzed *in vitro* for their magnetic properties. The relaxivity values (*r*_1_) of all contrast agents tested in the study are shown in Table 1. The measured values of all contrast agents were 3–35x higher than those of commercially available contrast agents under similar conditions (temperature, MRI scanner) (Table 1) (30, 32). When comparing the *r*_1_ of the different novel contrast agents with each other, PLA/HSA-DTPA-Gd (0.13 mM) and HSA-DTPA-Gd (0.06 mM) had the highest value with 116.0 and 96.8 mM^−1^ s^−1^, respectively. The chitosan-based contrast agents demonstrated high relaxivity values of 59.2 and 12.1 mM^−1^ s^−1^ corresponding to 0.076 and 3.00 mM, thus also surpassing commercially available contrast agents.

**Table 1:** List of contrast agents with their respective gadolinium concentrations and relaxivity values.

	**Contrast agent**	**Gd concentration, mM**	**Relaxivity *(r_**1**_)*,**
			**mM^**−1**^ s^**−1**^**
1.	HSA-DTPA-Gd	0.06	96.8
2.	HSA-DTPA-Gd	1.72	N/A
3.	PLA/HSA-DTPA-Gd	0.13	116.0
4.	Chitosan-DTPA-Gd	0.076	59.2
5.	Chitosan-DTPA-Gd	3.00	12.1

### *Ex vivo* MRI Measurements

All three MR compounds (HSA-DTPA-Gd, chitosan-DTPA-Gd and PLA/HSA-DTPA-Gd) showed signal enhancement in the sciatic nerve on the 7th day after surgery. For all nerves labeled with each of the prepared contrast agents, signal enhancement was visible in multiple consecutive cross sections. In the control group (*n* = 2), where prohance 25 mM was applied, no signal enhancement was identified along the sciatic nerve.

Spinal cord samples demonstrated a heterogeneous signal enhancement pattern on the 7th day after surgery. Focal signal enhancement was detected in the L4 spinal nerve at the level of the intervertebral foramen after application of HSA-DTPA-Gd 1.72 mM (*n* = 4). The corresponding T1 map revealed a T1 relaxation time of under 800 ms. Circumferential and diffuse signal alterations surrounding the spinal cord were observed after PLA/HSA-DTPA-Gd 0.13 mM (*n* = 2) or HSA-DTPA-Gd 0.06 mM (*n* = 2) application. Signal enhancement was also identified in the L5 spinal nerve after application of PLA/HSA-DTPA-Gd 0.13 mM (*n* = 1). Following chitosan-DTPA-Gd 0.076 mM application (*n* = 2), no signal enhancement could be seen in any spinal cord sample.

### *In vivo* MRI Measurements

**Figure 4:**
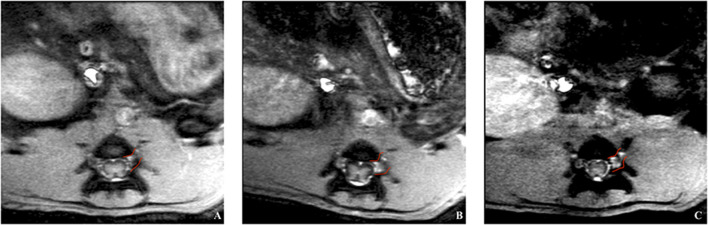
*In vivo* trial of a rat's spinal cord after application of chitosan-DTPA-Gd. Coronal MRI scans were acquired on the 1st **(A)**, 3rd **(B)** and 7th **(C)** day after surgery. The spinal nerve L5 is marked in red and the highest signal enhancement is identified on the 3rd day after the surgery, what corresponded with quantification of the signal enhancement (Figure 5B).

**Figure 5:**
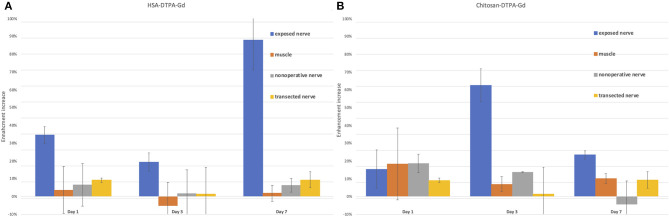
Quantification of signal enhancement increase in the sciatic nerve after HSA-DTPA-Gd **(A)** and chitosan-DTPA-Gd **(B)** application in various tissues. **(A)** The highest signal enhancement increase (88%) in the nerve was identified on the 7th day after the application of HSA-DTPA-Gd and was statistically significant compared to muscle (2%) and the native contralateral nerve (6%) (*p* < 0.05). **(B)** The enhancement increase was the highest (63%) on the 3rd day after application of chitosan-DTPA-Gd and statistically significant compared with unaffected muscle tissue (7%) and the native contralateral nerve (1%) (*p* < 0.05). Error bars represent standard deviation of the average values.

First, the native spinal cord and sciatic nerve were successfully visualized with axial and coronal T2WI sequences 1 day before the application of contrast agents (Figure 3). Signal enhancement was then detected in the spinal nerves on the 3rd and 7th, however, not on the 1st day after application of chitosan-DTPA-Gd 0.076 mM (Figure 4). Signal enhancement in the sciatic nerve (63%; 23%) increased statistically significant on the 3rd and 7th postoperative day compared with unaffected muscle tissue (7%; 10%) and the native contralateral nerve (1%; 9%), respectively, *(p* < 0.05) (Figure 5B). Signal enhancement increase was only statistically significant on the 3rd postoperative day (2%) when compared with the control group of rats with transected nerves without contrast agents *(p* < 0.05).

**Figure 6:**
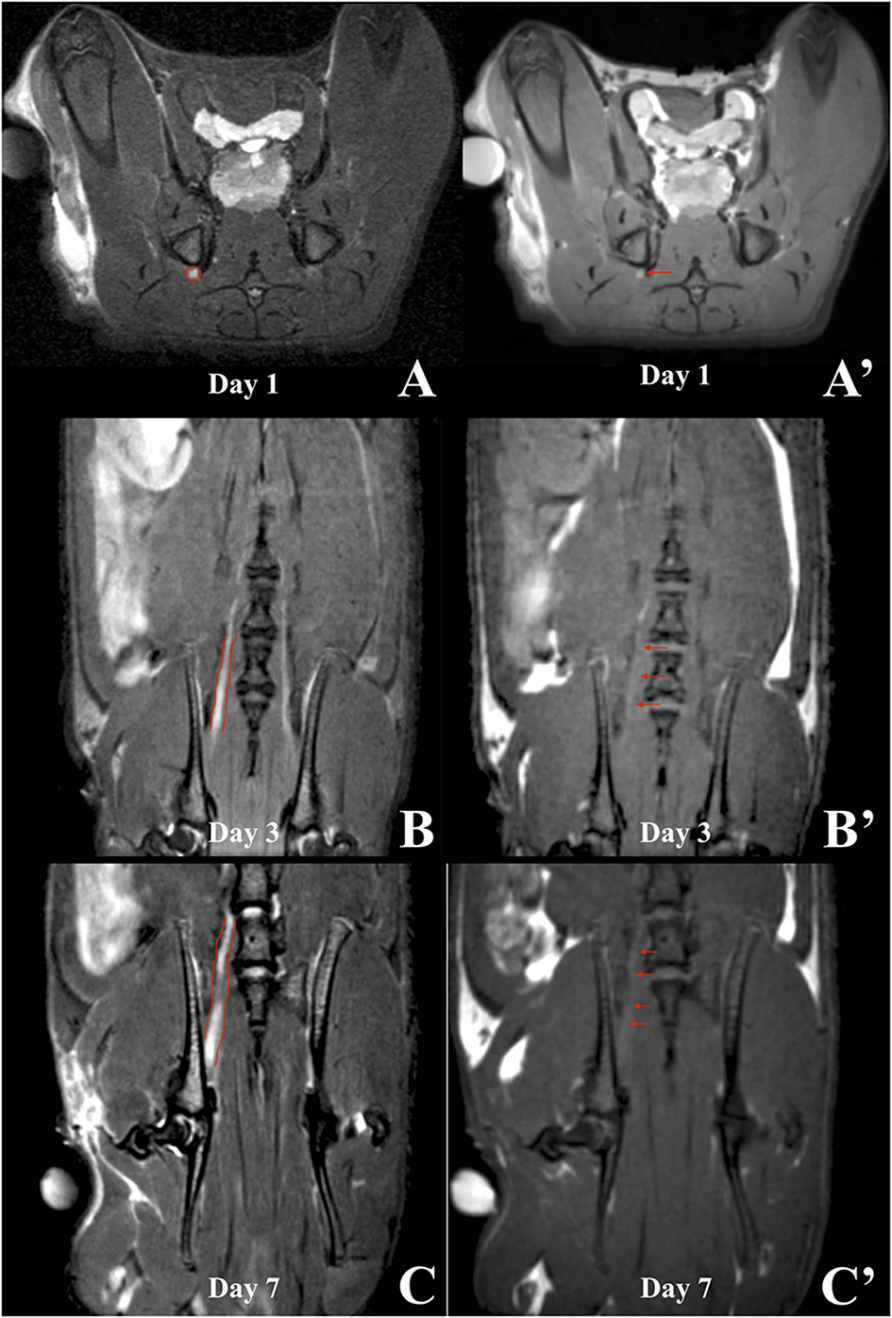
MRI scans of a rat in which HSA-DTPA-Gd was applied. T2WIs (left column) were compared to the corresponding T1WIs (right column) on the 1st **(A)**, 3rd **(B)** and 7th **(C)** day after surgery. Slight signal enhancement was visible on the 1st day (**A'**, red arrow) anatomically corresponding to the sciatic nerve in the T2WI (A, red circle). Coronal sections on the 3rd day after surgery did not show sufficient signal enhancement (**B'**, red arrows). On the 7th day, signal enhancement was shown along the proximal segment of the sciatic nerve distal to the intervertebral foramina (**C'**, red arrows), which corresponded to the anatomical course of the sciatic nerve and axonal transport of the contrast agent **(C)**.

The application of HSA-DTPA-Gd 0.06 mM led to an identifiable propagation of the signal enhancement along the proximal stump of the sciatic nerve after surgery (Figure 6). However, signal enhancement increase in the sciatic nerve (88%) was statistically significant on the 7th postoperative day compared with muscle tissue (2%) and the native contralateral nerve (6%) *(p* < 0.05). Compared to the control group with transected nerves, signal enhancement increase was statistically significant on the 1st and 3rd day (34 and 19% vs. 9 and 1%) (*p* < 0.05) (Figure 5A).

In summary, two of the three contrast agents tested in this trial showed signal enhancement in the sciatic nerve on the 3rd and 7th day after surgery: chitosan-DTPA-Gd 0.076 mM (*n* = 2) and HSA-DTPA-Gd 0.06 mM (*n* = 2). Application of PLA/HSA-DTPA-Gd 0.13 mM led to no detectable signal enhancement.

### Chemical Analysis

**Figure 7:**
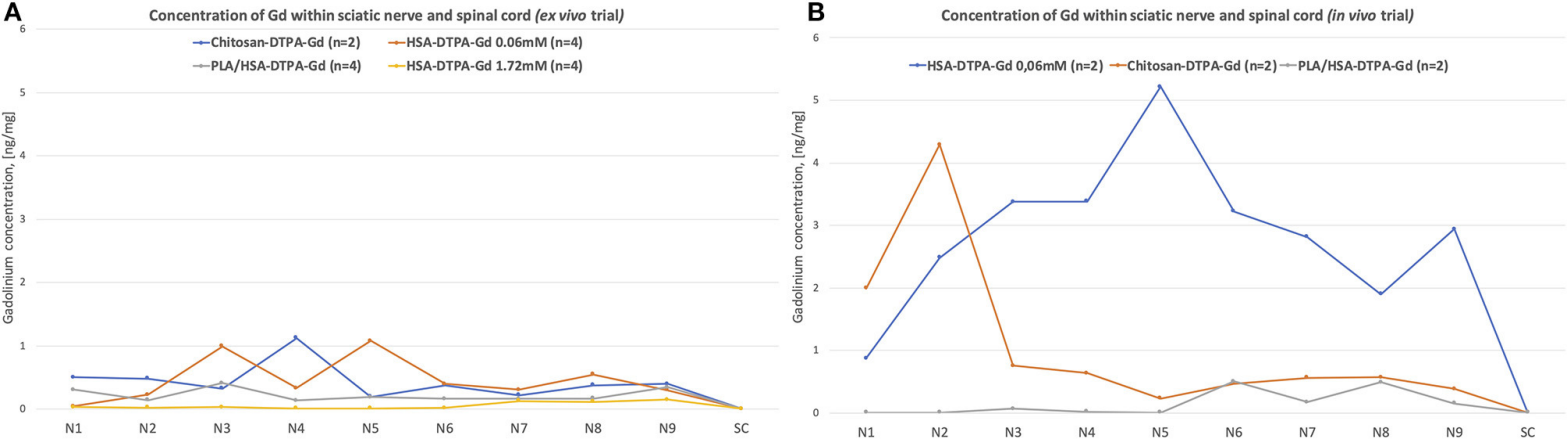
Chemical evaluation of Gadolinium concentration in the sciatic nerve and spinal cord from the *ex vivo* **(A)** and *in vivo* **(B)** trials. The nerve was cut into 50 mm segments, as indicated in the Figure 1, N1 corresponds to the most distal segment of the sciatic nerve, N2–N6 are more proximal segments of the nerve, N7, N8, and N9 correspond to the L6, L5, and L4 spinal nerves, respectively. Highest Gadolinium concertation (ng/mg) (1.078 ± 0.302 ng/mg in segment N5 and 1.197 ± 0.359 ng/mg in segment N4) was identified in *ex vivo* trial after application of HSA-DTPA-Gd 0.06 mM and chitosan-DTPA-Gd 0.076 mM, respectively **(A)**. In *in vivo* trial the highest Gadolinium concentration was identified after application of HSA-DTPA-Gd 0.06 mM (3.373 ± 0.053 to 5.217 ± 0.860 ng/mg in segments N3–N6) **(B)**. The Gadolinium concentration in in the spinal cord (SC) was negligibly low in all rats. High Gadolinium concentration along the sciatic nerve indicated successful uptake and propagation of contrast agents.

The radiological evidence was further verified by chemically assessing the temporospatial distribution of the contrast agents along the sciatic nerves. The highest Gd concentration was measured 1–2 cm proximal to the trifurcation of the sciatic nerve after applying HSA-DTPA-Gd 0.06 mM and chitosan-DTPA-Gd 0.076 mM with 1.078 ± 0.302 ng/mg in segment N5 and 1.197 ± 0.359 ng/mg in segment N4, respectively (Figure 7A). The contrast agents showed only low Gd concentrations ranging from 0.300 ± 0.211 to 0.990 ± 0.1743 ng/mg in the other nerve segments. The other contrast agents (PLA/HSA-DTPA-Gd, 0.13 mM and HSA-DTPA-Gd, 1.72 mM) showed only low Gd content ranging from 0.161 ± 0.029 to 0.011 ± 0.0219 ng/mg in all nerve segments (Figure 7A). Spinal cord samples showed negligibly low Gd concentrations for all tested contrast agents.

High Gd concentrations were measured in nerve samples from the *in vivo* trial following application of HSA-DTPA-Gd 0.06 mM varying from 3.373 ± 0.053 ng/mg to 5.217 ± 0.860 ng/mg in segments N3–N6 (Figure 7B). Chitosan-DTPA-Gd showed high Gd concentrations ranging from 4.291 ± 1.290 to 0.751 ± 0.701 ng/mg in the distal nerve segments (N1–N3). PLA/HSA-DTPA-Gd application led to very low Gd concentrations along the whole nerve (Figure 7B). Similar to the *ex vivo* trial, no quantifiable amount of Gd was measured in the spinal cord samples.

## Discussion

In this study, we present a novel approach for the visualization of peripheral nerves of rats using macromolecular MR contrast agents in completely new imaging setting. For the first time, we were able to demonstrate the temporospatial propagation of a contrast agent along a rat's sciatic nerve after targeted intraoperative application following MRI analysis. These novel findings allow the assessment of a nerve's course and functional continuity, which may aid in the evaluation of the level of a traumatic nerve lesion as well as the regeneration process after nerve reconstruction procedures.

Furthermore, this approach may be used in the radiological assessment of various nerve injuries and reconstruction models in rats. All prepared contrast agents showed superior relaxivity values than commercially available ones, resulting in better radiological efficacy (30). In the *ex vivo* trial, signal enhancement was demonstrated along the harvested sciatic nerve on the 7th day after application of PLA/HSA-DTPA-Gd 0.13 mM, chitosan-DTPA-Gd 0.076 mM and HSA-DTPA-Gd 0.06 mM when compared to nerves exposed to prohance or the native contralateral nerves. This showed, that the conventional Gd-based contrast agents necessitate concomitant coupling of a specific carrier molecule to facilitate retrograde transport along the nerve. *In vivo* measurements revealed signal enhancement along the sciatic nerve on the 3rd and 7th day after application of HSA-DTPA-Gd 0.06 mM and chitosan-DTPA-Gd. The radiological evidence was further supported by the Gd concentrations within the sciatic nerves measured in chemical *post-mortem* analyses, which suggested contrast agent accumulation as the basis of the signal enhancement.

As outlined above, the current clinical approach for assessing peripheral nerve injuries is based mainly on clinical and electrophysiological examinations (33). In clinical practice, however, many circumstances (swelling, muscles or nerves, that are anatomically difficult to access; or early posttraumatic phase) may reduce the reliability of such examinations (34). Alternatively, MR neurography can be performed to detect a nerve lesion regardless of the anatomical localization and assist in the evaluation of postoperative outcomes (10, 35, 36). Despite its wide applicability, MR neurography is susceptible to imaging artifacts, lacks specificity in T2WI, and, most notably, does not provide crucial data on axonal transport and thus functional continuity of axons (8, 37). In order to optimize signal specificity of lesioned nerves, various studies investigated contrast agent-enhanced MR neurography (13–21). Here, MR imaging was performed after systemic application of Gadofluorine M in rats with induced autoimmune neuritis, focal demyelination processes or crush injuries of the sciatic nerve. These studies demonstrated a correlation between Gadofluorine M-enhanced signals and nerve regeneration progress (13, 14, 19, 20). Different authors provided histological evidence of Gadofluorine M as well as superparamagnetic iron oxide particles bound to myelin debris accumulated within migrated macrophages (16, 20). Gadofluorine M is able to infiltrate regenerating nerve tissue due to increased permeability of the blood-nerve-barrier during Wallerian degeneration and demyelination processes (21, 38, 39). Therefore, signal enhancement after Gadofluorine M application only allows the visualization of the temporospatial process of nerve regeneration, mainly focusing on its inflammatory components. Interestingly, the aforementioned studies could not demonstrate signal enhancement of nerval structures after systemic application of Gd-DTPA in rats with ligation injury of the sciatic nerve, induced autoimmune neuritis or focal demyelination (13, 14, 19, 20, 34). On the other hand, Gd-DTPA-enhanced signals were demonstrated in the facial nerve following crush injuries in humans (40). Several studies demonstrated Gd-DTPA-enhanced signals in the median nerve of dogs and in the sciatic nerves of rats after crush injury (18, 21). These discrepancies may be explained by the different animal models, nerve injury mechanisms as well as MR imaging protocols. To prevent discrepancies in this novel proof-of-concept imaging approach, we did not use systemic administration of the contrast agent but used a well-established approach for retrograde axonal tracing of fluorescent dyes in the rat's sciatic nerve.

In this study we focused on the axonal transport as a driving force for contrast agents (41). Numerous factors influence transport direction and speed, yet we assume that for macromolecular contrast agents such as protein or polysaccharide-based compounds it is mainly their molecular structure and net charge (41). Therefore, we tested both larger (HSA-PLA-DTPA-Gd having a molecular weight over 200 kDa and an average nanoparticle size of about 163 nm; HSA-DTPA-Gd with ~78 kDa) and smaller (5 kDa chitosan, oligosaccharides) compounds as contrast agents for peripheral nerve imaging. We assume that the observed signal enhancement in the sciatic nerves on the third day after surgery in case of chitosan-DTPA-Gd and with HSA-DTPA-Gd on the seventh day is due to the different spreading velocities of these compounds or their cargo-complexes within the nerve.

Other novel diagnostic tools such as diffusion tensor imaging aim at delivering information about a nerve's functional integrity by distinguishing between its myelin sheath and axons (37). This imaging tool was further supported by experimental trials demonstrating a correlation between DTI-derived information and histological data of injured nerves (42, 43), as well as clinical trials comparing electrophysiological data with DTI-derived information in healthy patients (44) and patients with neuropathic changes (45). The major issue with interpreting DTI-derived tractography data to investigate a nerve's functional continuity lies in extrapolating data from water molecule movement in the extracellular space of peripheral nerve lesions. However, this only represents an indirect representation of axonal transport and subsequently nerve anatomy and function (46). Moreover, the integration of DTI into the routine clinical practice of MR neurography is limited by the scarce technical availability and high demand on resources (37).

In this study, contrast agents were successfully transported along the sciatic nerve, but no signal enhancement or chemical evidence were detected within the spinal cord (Figure 7). *Ex vivo* MRI scans showed a diffuse signal enhancement pattern within the vertebral cavity after application of HSA-DTPA-Gd and PLA/HSA-DTPA-Gd. The circumferential signal enhancement distribution around the spinal cord suggests an accumulation of contrast agents within the cerebrospinal fluid (CSF). As mentioned before, this may be explained by a disruption of the blood-nerve-barrier following nerve injury (21, 38, 39). Moreover, the multifocal appearance of contrast agents within the CSF may be explained by a communicating pathway, the so-called meningeal lip, between a peripheral nerve and the subarachnoid space, which was extensively described by Himango et al. in the rat model (47). This pathway may explain how the contrast agents in our study reached the CSF. Our findings correspond with a study by Chen et al., which demonstrated convection-enhanced transport of Gd-DTPA-albumin along the sciatic nerve into the CSF (48) using convection-enhanced delivery. In our study, we achieved equivalent results using an alternative delivery mechanism. Notably, we utilized passive delivery without exerting pressure on the sciatic nerve to infuse higher volumes (12–47μl) of Gd-DTPA-albumin into it. Passive transport of contrast agents may present a more gentle, precise and efficient delivery method, preventing leakage from spinal nerves or damaged connective tissue of the nerve. Overall, the true nature of the transport mechanism remains unclear. According to Chen et al., Gd-DTPA-albumin was considered to be transported by convection and diffusion mechanisms using convection-enhanced delivery (48).

A limitation of this proof-of-concept study is the small sample size of the trial with different contrast agents. Nevertheless, the statistically significant data from the *in vivo* trial still highlighted successful transport of contrast agents. MRI measurements were performed within 1 week on the 1st, 3rd, and 7th day after contrast agent application. Measurements done beyond 1 week may provide additional insights into the dynamics of contrast agent propagation in future studies. Additionally, future studies may investigate imaging of other nerve injury types (e.g., traction or crush injury) or to evaluate the success of surgical reconstruction. In this regard, intramuscular or intradermal application of contrast agents may also be interesting to evaluate the connection between muscles or skin and their innervating nerves (25). Thus, we assume our novel imaging modality may be used to visualize all types of peripheral nerves (sensory, motor or mixed) and different nerve injury types using less traumatic application of contrast agents (e.g., intramuscular, intraneural or epineural application). Thus, it is essential to conduct further trials with bigger sample size to investigate the transport mechanism behind our novel approach and long-term outcomes.

In this study we established for the first time a novel MRI imaging modality for peripheral nerves using retrograde transport of contrast agents along the nerve. This novel approach to contrast agent delivery along peripheral nerves has a wide spectrum of potential experimental and clinical applications. Using this approach, one may be able to determine functional continuity of peripheral nerves after injury or reconstruction. Therefore, it could be applied in various experimental models for nerve regeneration. Since imaging modalities for peripheral nerve lesions are limited, visualization of functional integrity of axonal transport using MRI may be a useful tool to diagnose and precisely localize nerve lesions. Moreover, retrograde transport of our molecular complexes up to the CSF may provide a targeted drug delivery mechanism for neurodegenerative diseases or traumatic nerve injuries.

## Data Availability Statement

The raw data supporting the conclusions of this article will be made available by the authors, without undue reservation.

## Ethics Statement

The animal study was reviewed and approved by Austrian Federal Ministry of Education, Science and Research.

## Author Contributions

VT, KM-S, IP-M, JF, KB, MA, JK, BK, TH, and OA: conception and design. VT, KM-S, CF, KB, MA, and OA: surgical procedure. VT, KM-S, JF, ST, KB, TH, and OA: imaging analysis. IP-M, ST, MK, and BK: chemical analysis. VT, JF, KM-S, IP-M, CF, JK, TH, and OA: statistical analysis. VT, KM-S, IP-M, JF, LH, JK, KB, BK, TH, and OA: assembly and analysis of data. VT, KM-S, IP-M, JF, CF, KB, LH, JK, BK, TH, and OA: drafting of the article. All authors contributed to the article and approved the submitted version.

## Conflict of Interest

The authors declare that the research was conducted in the absence of any commercial or financial relationships that could be construed as a potential conflict of interest.
